# Need for a Dedicated Ophthalmic Malignancy Clinico-Biological Biobank: The Nice Ocular MAlignancy (NOMA) Biobank

**DOI:** 10.3390/cancers15082372

**Published:** 2023-04-19

**Authors:** Arnaud Martel, Lauris Gastaud, Christelle Bonnetaud, Sacha Nahon-Esteve, Kevin Washetine, Olivier Bordone, Myriam Salah, Virginie Tanga, Julien Fayada, Virginie Lespinet, Maryline Allegra, Salome Lalvee, Katia Zahaf, Stephanie Baillif, Corine Bertolotto, Baharia Mograbi, Sandra Lassalle, Paul Hofman

**Affiliations:** 1 Ophthalmology Department, Nice University Hospital, 06001 Nice, France; 2 Institute of Research on Cancer and Aging in Nice (IRCAN), Team 4, Centre Antoine Lacassagne, Centre National de la Recherche Scientifique (CNRS), Institut National de la Santé et de la Recherche Médicale (INSERM), Fédération Hospitalo-Universitaire (FHU) OncoAge, Côte d’Azur University, 06189 Nice, France; 3 Oncology Department, Antoine Lacassagne Cancer Centre, 06000 Nice, France; 4 Hospital-Integrated Biobank (BB-0033-00025), Centre Hospitalier Universitaire de Nice, Fédération Hospitalo-Universitaire (FHU) OncoAge, Côte d’Azur University, CEDEX 1, 06001 Nice, France; 5 Laboratory of Clinical and Experimental Pathology, Centre Hospitalier Universitaire de Nice, Fédération Hospitalo-Universitaire (FHU) OncoAge, Côte d’Azur University, 06000 Nice, France; 6 C3M, Institut National de la Santé et de la Recherche Médicale (INSERM), Côte d’Azur University, 06200 Nice, France

**Keywords:** biobank, ophthalmic malignancies, uveal melanoma, tissue biopsy, liquid biopsy database

## Abstract

**Simple Summary:**

Ophthalmic malignancies refer to rare, highly diversified, aggressive neoplasms. Unlike other types of solid tumors, tissue biopsy is not recommended for intraocular malignancies so a limited number of tissue samples are available for translational research programs. To date, very few biobanks dedicated to ophthalmic malignancies have been reported. The aim of this article was to present the challenges raised by ophthalmic malignancies in order to obtain biospecimens for research purposes, and the biobank dedicated to ophthalmic malignancies set up in our institution (Côte d’Azur University, Nice, France) was detailed. There is an urgent need to develop ocular malignancy biobanks to enhance translational research projects, develop international collaborations, and, ultimately, optimize customized medicine for the treatment of these tumors.

**Abstract:**

Ophthalmic malignancies include various rare neoplasms involving the conjunctiva, the uvea, or the periocular area. These tumors are characterized by their scarcity as well as their histological, and sometimes genetic, diversity. Uveal melanoma (UM) is the most common primary intraocular malignancy. UM raises three main challenges highlighting the specificity of ophthalmic malignancies. First, UM is a very rare malignancy with an estimated incidence of 6 cases per million inhabitants. Second, tissue biopsy is not routinely recommended due to the risk of extraocular dissemination. Third, UM is an aggressive cancer because it is estimated that about 50% of patients will experience metastatic spread without any curative treatment available at this stage. These challenges better explain the two main objectives in the creation of a dedicated UM biobank. First, collecting UM samples is essential due to tissue scarcity. Second, large-scale translational research programs based on stored human samples will help to better determine UM pathogenesis with the aim of identifying new biomarkers, allowing for early diagnosis and new targeted treatment modalities. Other periocular malignancies, such as conjunctival melanomas or orbital malignancies, also raise specific concerns. In this context, the number of biobanks worldwide dedicated to ocular malignancies is very limited. The aims of this article were (i) to describe the specific challenges raised by a dedicated ocular malignancy biobank, (ii) to report our experience in setting up such a biobank, and (iii) to discuss future perspectives in this field.

## 1. Introduction

Ophthalmic malignancies include a wide spectrum of highly variable tumors involving the conjunctiva, the uvea, the eyelids, and the orbit. Most of these tumors are considered rare and there is no consensus standard of care [1]. Uveal melanoma (UM) is the most common primary intraocular malignancy, with an incidence of only 6 cases per million inhabitants in Western countries [2]. Therefore, UM is considered a rare but aggressive malignancy [3]. The last decade has been marked by dramatic molecular and genetic improvements related to UM. However, the daily clinical management of patients is lacking and it is estimated that 50% of UM patients will experience metastatic spread [4]. To date, there is no curative treatment available for metastatic UM. Current animal models are scarce, especially metastatic models, and these do not allow for the generalization of the findings to humans [5]. Therefore, there is an urgent need to develop customized targeted treatments through collaborative translational and clinical research based on robust clinical, imaging, biological, and genetic data obtained from patients [6].

In 1996, the term “biobank” was first introduced and rapidly popularized [7]. Most human malignancies now have dedicated biobanks [8]. However, ophthalmic malignancy biobanks are poorly reported in the literature, thus limiting data sharing and international collaboration [9]. Since 2013, the Laboratory of Clinical and Experimental Pathology of Nice University (France) has maintained an accredited biobank dedicated to ophthalmic malignancies that belongs to the Cote d’Azur Biobank (BB-0033-00025, https://biobank-cotedazur.fr, accessed on 1 January 2023). 

The aims of this article were to detail the Nice Ophthalmic MAligancy (NOMA) biobank and to highlight the current challenges, objectives, and limitations encountered when setting up such a dedicated ophthalmic malignancy biobank.

## 2. What Are the Specific Challenges Associated with Ophthalmic Malignancies?

### 2.1. A Wide Variety of Malignancies

**Figure 1:**
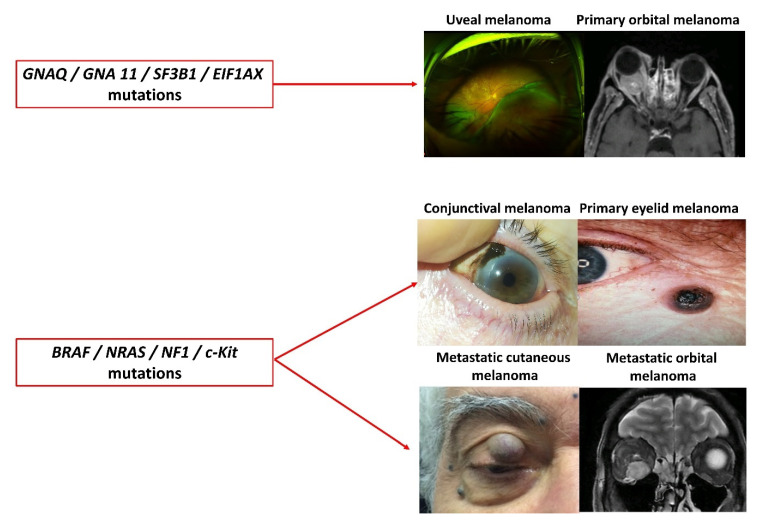
The spectacular variety of periocular melanomas.

The periocular area is a unique and exceptional example of melanoma diversity in the human body. Several types of melanomas may be found in clinical practice, such as primary or metastatic cutaneous melanomas, conjunctival melanomas (CM), UM, and primary or secondary orbital melanomas (Figure 1). For example, although anatomically close (only a few millimeters), CM and UM are very different genetically [10]. It is now well-established that UM and primary orbital melanoma result from driver mutations in the *GNAQ* and *GNA11* genes, whereas cutaneous melanoma and CM result from mutations in the *BRAF*, *NRAS*, *NF1*, and *c-Kit* genes [11]. These molecular differences explain why CM has benefited from the therapeutic opportunities developed for cutaneous melanoma with anti-BRAF and anti-MEK targeted therapies unlike UM [1,12]. Another major difference is the role of the immune system in UM, CM, and cutaneous melanoma. Due to the “immune privilege”, most conventional anti-PD-1 and anti-CTLA-4 therapies have failed to demonstrate any benefit in UM, while they are effective in cutaneous melanoma and CM [13]. Tebentafusp, a specific anti-GP100 immunotherapy, has recently shown promising results in metastatic UM but a longer follow-up is needed to ascertain its efficacy [14]. 

### 2.2. A Wide Variety of Clinical Presentations and Aggressiveness

In line with other tumors located elsewhere, the clinical presentation may vary greatly from one patient to another. A few decades ago, the prognosis of UM was essentially based on histological findings, and the epithelioid subtype, a high mitotic index, or an extraocular extent were considered poor prognostic factors [15]. More recently, the loss of expression of BAP1 has been shown to be a major pejorative prognostic factor through the induction of tumor dissemination in UM [16] and it is now systematically investigated in daily clinical practice [17]. Lately, chromosomal and genetic findings have revolutionized the prognosis of UM patients by allowing for the distinguishing of patients with a low versus high metastatic risk [16]. Monosomy of chromosome 3 associated with chromosome 8q gain has been shown to be highly predictive of metastatic spread by Trolet et al. [18]. Also, Onken et al. have shown that 15-gene expression profiling in UM allowed for the accurate distinction of class 1 (low risk) and class 2 (high risk) metastatic risk profiles [19]. 

### 2.3. Tumor Rarity

Except for eyelid basal cell carcinoma, most ophthalmic malignancies are extremely rare, thus limiting the collection of numerous tissue samples. The annual incidence per million inhabitants of several ophthalmic malignancies is summarized in Table 1. A slight increase in CM incidence has been found over the last few decades [20], but this has not been confirmed in UM [15,21].

### 2.4. Lack of Tissue Biopsy: The UM Example

Unlike most malignancies, the diagnosis of UM is based on clinical and ocular ultrasound findings with no need for a tissue biopsy. Transscleral or transvitreal UM biopsies are not recommended and are even contraindicated because they are considered unsafe (risk of intravitreal haemorrhage, retinal detachment) with a non-negligible risk of extraocular tumor dissemination [24]. The only interest of tissue biopsy would be to assess tumor prognosis. However, because there is no curative treatment for disseminated UM, this argument is widely discussed, and the benefit/risk balance of UM biopsy does not appear to be favorable. About 90% of UM cases will be treated conservatively with proton therapy, radiosurgery, or brachytherapy with favorable local outcomes [15]. Only large or diffuse intraocular disseminated tumors are treated with enucleation. The last decade has been marked by an increase in eye-sparing indications and even large UM are increasingly eligible for conservative treatments without major side effects [25]. Taken together, these data explain why UM samples are usually not collected to build biobanks.

### 2.5. Emergence of Liquid Biopsies

Because most ophthalmic malignancies are rare and since tissue biopsy is not routinely performed in these latter tumors, liquid biopsies (LBs) have recently been popularized [26]. LB in the onco-ophtalmology domain mainly refers to the collection of any potential tumor fluids, such as venous blood or aqueous humor (AH). Several features may be monitored, including circulating tumor cells (CTCs), circulating tumor DNA (ctDNA), circulating tumor RNA (ctRNA), circulating vesicles such as exosomes, and tumor-educated platelets [26]. The advantages of LB include: (i) its non-invasiveness, (ii) the fact that it may be repeated as often as required, thus allowing for disease monitoring, (iii) the large amounts of fluid available, (iv) the fact that it better reflects the metastatic spread of the disease in the case of venous LB, and (v) the fact that it allows the establishment of a reliable prognosis in various malignancies [26]. Thus, two types of LBs are currently being investigated in the field of ophthalmic malignancies. 

#### 2.5.1. Venous Liquid Biopsies: The UM Example

CTC and ctDNA have been studied in various prospective studies in UM. CTCs were first investigated in UM in 2008 by Ulmer et al. [27]. CTCs appear to be tumor-specific since they have never been identified in healthy patients and benign lesions. Higher CTC counts have been found in metastatic versus primary UM and in class 2 versus class 1 tumors [26]. It is still unclear whether CTC detection in primary UM might predict further metastatic spread. This feature is of strong interest because it could justify the use of systemic adjuvant treatment for the management of primary UM. The limitations include the use of a wide variety of CTC isolation and identification methods [26].

Higher ctDNA levels have also been found in metastatic UM compared to primary UM. Only one study has compared the CTC counts and ctDNA levels in 40 patients with metastatic UM, and CTCs were detected using the CellSearch^®^ (Menarini Silicon Biosystems, Florence, Italy) device in only 30% of patients, while the ctDNA was detected in 84% of patients [28]. Interestingly, only the ctDNA significantly correlated with the progression-free survival and overall survival in the multivariate analysis [28]. 

#### 2.5.2. Aqueous Humor (AH) Biopsies

AH collection is a simple, minimally invasive procedure usually performed on an outpatient basis. AH LBs have been mainly studied in retinoblastomas which are the most common primary intraocular malignancy found in childhood. The body of evidence indicates that AH collection is useful for the diagnosis and monitoring of treatment response [29]. It has been shown that AH collection is much more reliable than venous LB [30]. Only a few studies have investigated AH LBs in UM and they have suggested that the AH could be used as a diagnostic and prognostic biomarker in the future [31,32].

## 3. Legal, Economic, and Technical Aspects

### 3.1. Legal Considerations

The NOMA biobank is part of the hospital-integrated Cote d’Azur Biobank stored and managed by Nice University Hospital (France). The Cote d’Azur Biobank has developed several platforms, including tissue, LB, and molecular pathology platforms, located in the Laboratory of Clinical and Experimental Pathology (www.biobank-cotedazur.fr, accessed on 1 January 2023) [33]. The main tumors stored have been collected from patients with lung, thyroid, skin, head and neck, and more recently, ocular diseases. The Côte d’Azur Biobank obtained the NF S96-900 certification (National French Standard) in 2010. In 2022, it obtained the ISO 9001 certification as well as the international ISO 20387 standard specific for biobanking activity [34]. These standards guarantee the high quality of the whole biobanking process. Moreover, the LPCE has obtained an accreditation according to the ISO 15189 norm in 2013 for surgical and molecular pathology diagnosis, notably for ophtalmopathology, therefore ensuring the quality of the pre-analytical, analytical, and post-analytical steps before transferring samples into the Côte d’Azur Biobank.

### 3.2. Funding Considerations

A major issue regarding biobanks, especially biobanks dedicated to a specific pathology, is their financial viability in terms of development and long-term maintenance [9,35,36]. Creating a biobank requires an expert team, various costly processes and equipment, in particular for sample storage, along with computer software [33,37]. The business model of the Côte d’Azur Biobank (BB-0033-00025) is partially supported by French public funding programs funded by the French Ministry of Health, such as the MERRI (“Mission d’Enseignement, Recherche, Reference et Innovation”) and DGOS (“Direction Générale de l’Offre de Soins”) programs. To a lesser extent, different material transfer agreements (MTAs) with public and private institutes allow for biobank funding [33]. These public–public and public–private partnerships were included in the yearly Côte d’Azur Biobank budget [33,38]. In this regard, a costing policy for samples and various associated expertise provided by the biobankers to the scientists, and contracts and MTAs have been elucidated in association with the Research and Innovation Department of Nice University Hospital. These costs and their applications have been assessed according to national and international recommendations while taking into account the share of investment for the biobank and the scientific collaboration level [39]. The contracts signed by the scientists and other partners require mentioning the biobank in their publications in different sections depending on the contribution of the biobank members (co-author, citation of the biobank in the Acknowledgments section).

### 3.3. Technical, Space, and Computer Considerations

The implementation of the NOMA Biobank is summarized in Table 2. Tissue biopsy, liquid biopsy, and AH puncture were stored in the NOMA biobank in 2013, 2018, and 2020, respectively. 

#### 3.3.1. Biobanking Process for UM

**Figure 2:**
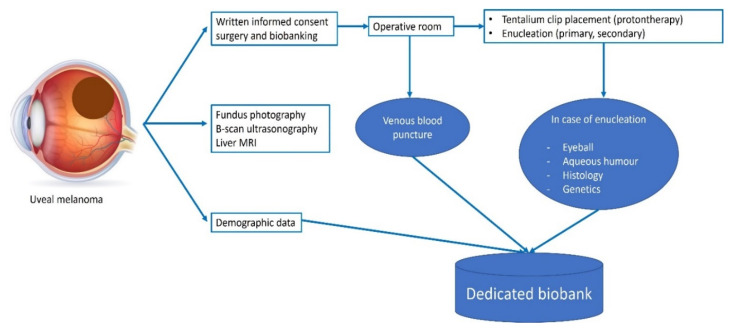
Whole biobanking process for UM patients.

The biobanking process, illustrated for UM, is summarized in Figure 2. Briefly, the diagnosis of UM is based on clinical and ultrasound findings obtained by ocular oncologists at Nice University Hospital, one of the two tertiary referral care centers in ocular oncology in France [40]. Preoperative examination includes dilated fundus examination, ultra-wide field retinography (Optomap, Optos PLC, Dunfermline, Scotland, UK), OCT, and ICG angiography, when required. A systemic work-up includes a liver MRI or ultrasonography. Written informed consent is signed preoperatively by the patients in order to be able to include them into the biobank database. 

In UM patients, undergoing conservative surgery (placement of tantalum clips followed by proton therapy) and venous LBs (4 EDTA blood collection tubes of 8 mL) may be performed at the time of initial surgery. We no longer perform transscleral biopsies due to their low yield and the risk of extraocular tumor dissemination. In patients undergoing primary or secondary enucleation, AH collection (once the eye has been enucleated), LB, tissue histology, and genetic analyses may be performed. The NOMA biobank and the LPCE are located in the same department. The operative rooms are closely connected to the laboratory with the use of an intra-hospital pneumatic tube transportation system. Solid and LB samples are usually processed within 15 min of receipt in the laboratory. The processing, amount, and storage conditions for each sample type are summarized in Table 3. 

Regarding the various procedures for tissue sample collection, the diagnosis was made and the specimens to be collected for the biobank were selected by a pathologist expert in ophthalmology (SL). When possible, the tumor tissue as well as the healthy adjacent tissue were both frozen or fixed in formalin and stored. For each tumor with frozen samples, a mirror sample was embedded in paraffin for quality control and assessment of the percentage of tumor cells, and one fragment was dedicated to the diagnosis while the others were stored for research purposes. Each tissue sample was weighted before freezing. All the samples were processed and stored in appropriate facilities in accordance with standard guidelines for equipment, computing, and safety. Clinical and imaging data are stored in a dedicated accredited database (Synapse medical, Table 4). Synapse software is a specific health data warehouse approved by the CNIL (Commission Nationale de l’Informatique et des Libertés; https://www.cnil.fr/, accessed on 1 January 2023). Biological, histological, and genetic data are saved in another dedicated software belonging to the Cote d’Azur Biobank (https://biobank-cotedazur.fr, accessed on 1 January 2023). We recently acquired MBioLIMS BIOBANKING^®^ software (Modul-bio, Marseille, France) specifically dedicated to biobanking activities. This allows for a complete view of all the samples stored and statistical reports on the biobank activity [42]. Trolet classification was established in 2009 based on tumoral chromosomal abnormalities [18]. Two subtypes based on chromosome 3 disomy (Trolet 1a, 1b) and chromosome 3 monosomy (Trolet 2a, 2b, 2c) have been established. Gain of 8q associated with monosomy 3 was classified as Trolet 2b or 2c and it has been strongly associated with an increased risk of metastatic spread [18,43]. In this work, tumoral DNA extraction was performed on paraffin tissue sections using the Maxwell automate (Promega, Madison, WI, USA) with a dedicated kit. Genomic hybridation and nucleotidic polymorphisms were established by using CGH-array and SNP-array analyses with the Genechip Oncoscan Array (Affymetrix, Santa Clara, CA, USA).

#### 3.3.2. Process for Other Ophthalmic Malignancies

The storage process for other ophthalmic malignancies is similar to that described above. Table 5 summarizes the main data recorded for other ophthalmic malignancies.

## 4. Resources of the NOMA Biobank

### 4.1. All Tumors

**Figure 3:**
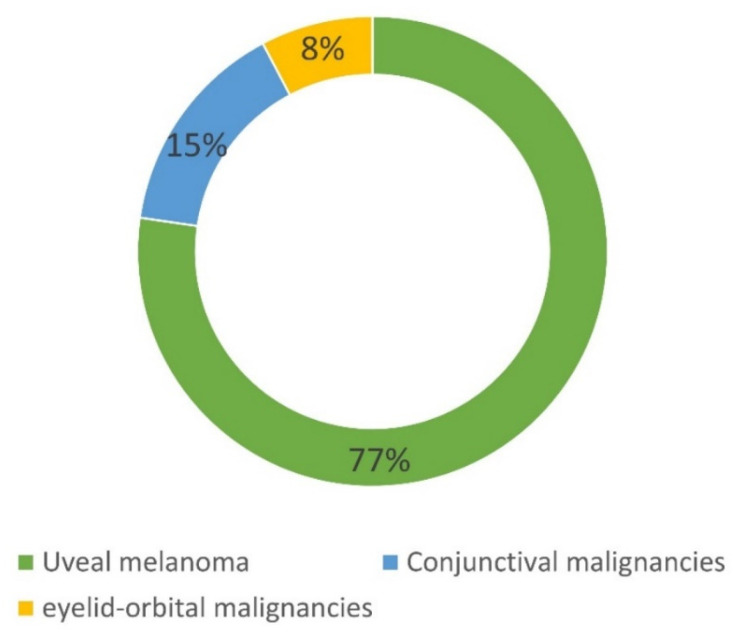
Composition of the NOMA Biobank samples.

Overall, 207 patients have been included in the NOMA biobank since 2013. As shown in Figure 3, 160 patients had an UM, 31 had a CM, and 16 had an orbital malignancy. Table 6 summarizes the main histological subtypes of the tumors stored in the NOMA biobank. For the 160 patients with an UM, 41% and 77.5% of samples were stored as LBs and solid biopsies, respectively. This preponderance of LB samples is easily explained by the fact that tissue biopsy is not routinely performed in the case of UM and is only obtained when enucleation is performed. The low number of conjunctival and orbital tumors included in the NOMA Biobank is explained by the fact that the tumor size is usually too small to be able to include tissues in a biobank and the samples are only used for clinical diagnostic purposes.

### 4.2. Uveal Melanoma

**Figure 4:**
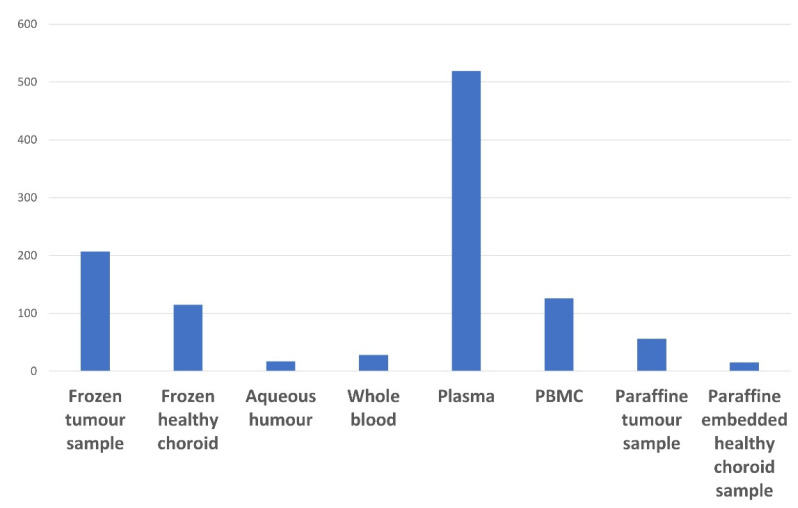
Summary of the UM tissue and liquid biopsy samples available in the NOMA Biobank.

The number of UM samples available in the NOMA Biobank is summarized in Figure 4. Regarding LBs, most samples were prepared and stored as plasma samples. Only a few AH samples (15 patients) were available in our biobank. This is explained by the fact that we have only systematically collected the AH from enucleated eyeballs since 2020.

**Figure 5:**
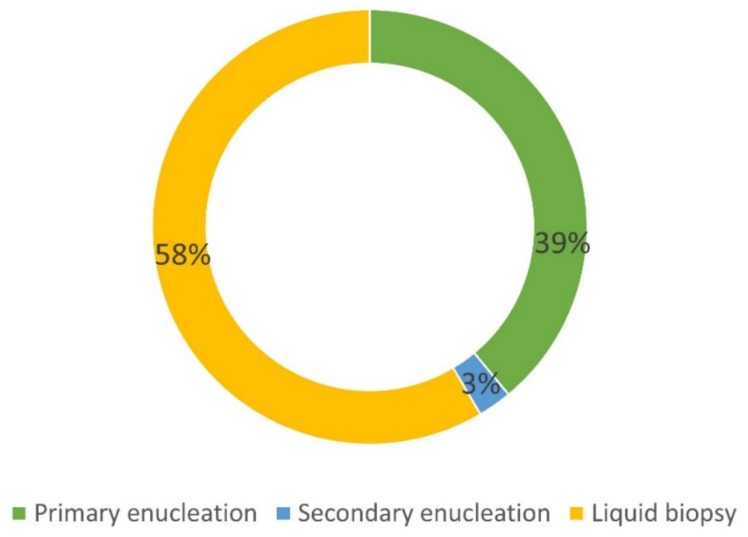
Details of the origin of UM samples included in the NOMA Biobank. Primary enucleation: no treatment before surgery. Secondary enucleation: treatment before surgery (proton therapy).

Among the 160 UM patients included in the NOMA Biobank, 66 (41%) were enucleated, thus allowing for tissue collection (Figure 5). 

The histological features of the enucleated eyeballs are provided in Table 7. Among the patients who underwent secondary enucleation, only those referred for tumor recurrence were included. Patients who underwent secondary enucleation for painful and blind eye due to extensive necrosis of the tumor with proton therapy were not included in the biobank. The cold ischemia time was defined as the time between enucleation and sample freezing for the biobank. The BAP 1 status was assessed by immunohistochemistry. Most of the UM tumors showed histological signs of aggressiveness (67% of cases with epithelioid cells, 20% of cases with extraocular extent, 33% of cases with ciliary body involvement). 

**Figure 6:**
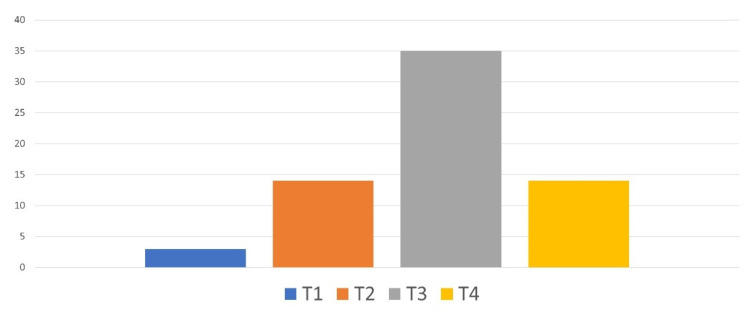
Pathological TNM classification of the tumors of enucleated patients.

The pathological Tumor–Node–Metastasis (pTNM) classification of the tumors of enucleated patients is provided in Figure 6. About half of the patients had a pT3 UM. 

**Figure 7:**
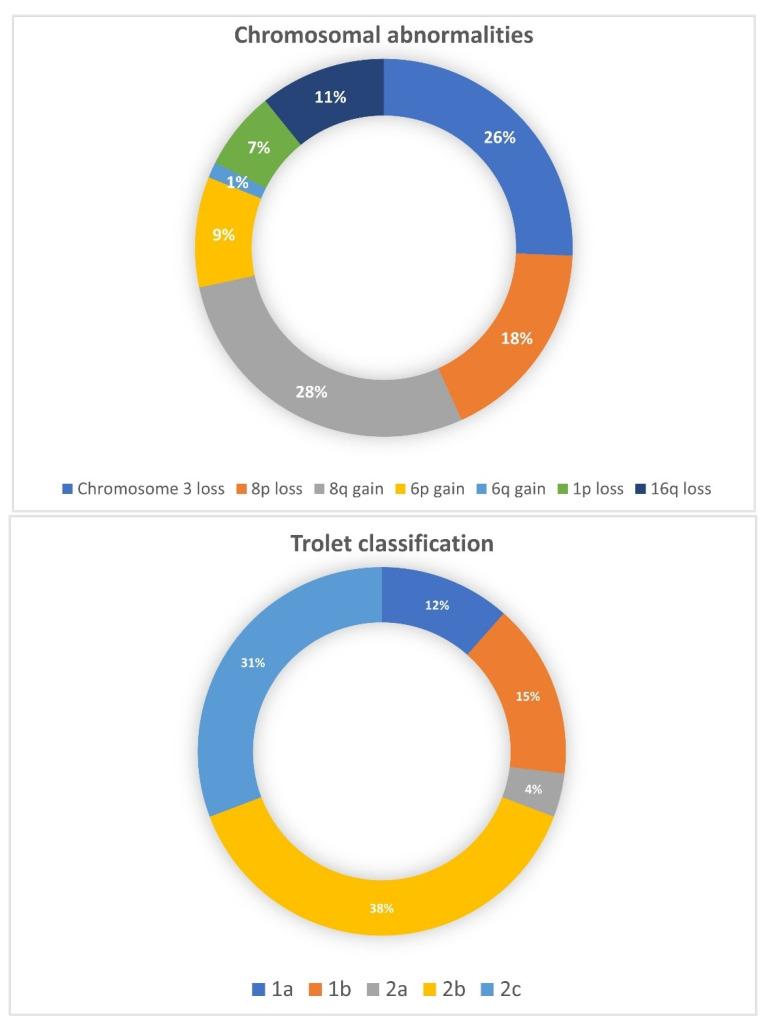
Chromosomal abnormalities and Trolet classification of 28 enucleated UM patients.

Among the 66 enucleated patients, genetic analyses were performed in 28 (42%) patients. The chromosomal abnormalities as well as the subsequent Trolet classification are shown in Figure 7. Overall, most patients were classified as “poor prognosis” with the loss of a chromosome 3 and a chromosome 8q gain and 64% were classified as “Trolet 2b and 2c”. These findings were consistent with the histological features described above.

## 5. Objectives of Setting Up a Dedicated Ophthalmic Malignancy Biobank

### 5.1. Diagnosis

The diagnosis of a conjunctival or periocular malignancy is routinely made by pathologists. However, in certain circumstances such as UM, a tissue biopsy is not recommended, and treatment is only based on clinical and radiological findings, incurring a risk of diagnostic inaccuracy, such as the enucleation of a non-tumor intraocular mass. AH collection is not routinely performed for the diagnosis of UM. However, it is a minimally invasive procedure performed on an outpatient basis. Several studies have recently shown that the presence of driver mutations in the *GNAQ* and *GNA11* genes could be used as a reliable diagnostic biomarker in UM, thus highlighting the need for a dedicated venous blood and AH biobank in UM [32].

### 5.2. Translational Research and Precision Medicine

Healthy and tumor tissues as well as LBs from ocular tumors are stored in our biobank, allowing translational research and tumorigenesis exploration. As highlighted above, very rare samples, such as AH samples, are systematically collected prospectively. AH samples should permit the identification of biomarkers for the diagnosis of UM and retinoblastoma follow-up [31]. To date, no targeted therapy has been found to be effective in the treatment of metastatic UM nor the prevention of metastatic spread [12]. In 2018, the tyrosine kinase inhibitor sunitinib administrated in adjuvant therapy in high risk (loss of chromosome 3, gain 8q, Class 2, T3–T4 by American Joint Committee on Cancer classification) primary UM was considered promising due to improvements in overall survival [44]. However, the study was not randomized (since the control group was an historical cohort) and results were not subsequently validated in an independent cohort. However, this study highlights the urgent need to discover new targeted therapies that can be administered, and make room for adjuvant therapeutic decision-making during the primary treatment of ocular malignant tumors. In this context, the systematic storage of UM samples exhibits a strong value for translational research by identifying future therapeutic targets.

### 5.3. Impact of Radiotherapy on UM Genetics

The storing of secondary enucleation samples following radiotherapy is of high interest and has been minimally studied until now. The impact of radiotherapy on genetic abnormalities and subsequent prognosis is still debated since controversial studies on this topic have been reported. Genetic abnormalities have been found to be more prevalent and more complex in previously irradiated tumors [45]. In contrast, another study found that chromosome 3 status and subsequent prognosis were not modified by radiotherapy [46]. There is also a debate regarding the difficulty of assessing genetic status (especially chromosome 3) in previously irradiated UM. Furthermore, karyotyping and FISH detection were found to be significantly altered by radiotherapy [45], whereas in another study, the detection of chromosome 3 microsatellite analysis was successfully determined [47]. In our experience, genetic testing depends on the etiology of secondary enucleation. Secondary enucleations performed for blind and/or painful eyes are associated with a high rate of post-radiotherapy necrosis, leading genetic testing to be inconclusive. Conversely, secondary enucleation performed for tumor recurrence can be more easily processed. However, it is not always clear whether genetic testing in this case is carried out on a recurrent tumor (i.e., tumor resistance to radiotherapy) or on a primary tumor located outside the field of irradiation. 

### 5.4. Pretreatment Screening

A biobank may also be useful to investigate whether a patient might be eligible for a given treatment. Until recently, there was not standard of care for metastatic UM. Tebentafusp, a specific anti-GP100 immunotherapy, has recently been approved by the Food and Drug Administration but only in HLA-A*02:01 UM patients [48]. The HLA status of patients with cryopreserved blood samples can be easily determined and might help clinicians determine whether or not a patient is a good candidate for tebentafusp treatment. 

### 5.5. Scientific Output

**Figure 8:**
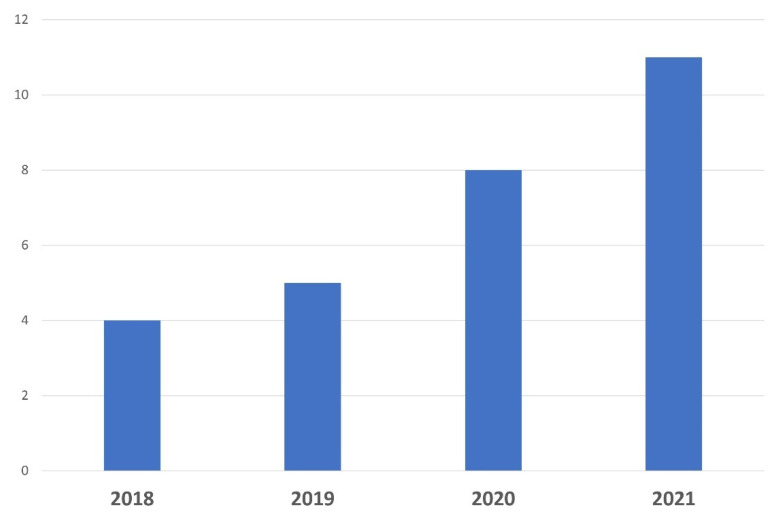
Number of scientific publications related to ocular oncology and translational research published over the last 4 years by Nice University Hospital thanks to the use of samples.

A biobank dedicated to a specific pathology should be managed by an expert team involved in the setting up and development of research projects in tight collaboration with physicians, surgical and molecular pathologists, and researchers. Thus, a specific biobank should be acknowledged and cited in scientific publications. As shown in Figure 8, the number of publications related to ocular oncology published by our department has markedly increased over the last few years, both for fundamental and clinical research programs, mainly through the use of ocular resources from the biobank. Several articles based on the NOMA biobank resources have recently been published in peer-reviewed journals with high impact factors [1,6,49,50,51].

### 5.6. National and International Collaborations

Data sharing enabled Nice University Hospital to collaborate with several internationally recognized ocular oncology departments, as summarized in Table 8. Of the 369 FFPE samples stored in the NOMA biobank, 44 (11.9%) have been used for collaborative scientific projects. A specific scientific and ethics committee is mandated to assess each public or private request to use samples according to the current regulations.

### 5.7. Information for Patients and Other Health Professionals

Because clear and accurate information is mandatory before obtaining any written informed consent, our Ocular Oncology Department has recently developed a dedicated website for patients referred to our tertiary care center (https://www.cancerdesyeux.fr, accessed on 1 January 2023). This website provides valuable information on patients’ care and follow-up, as well as research projects. A dedicated website has also been created by the Clinical and Experimental Pathology Laboratory Department of Nice University Hospital to provide useful information to patients and health professionals (http://biobank-cotedazur.fr/, accessed on 1 January 2023). Scientific congresses and relevant learned societies such as the ISOO (International Society of Ocular Oncology), OOG (Ocular Oncology Group), ESOPRS (European Society of Ophthalmic Plastic and Reconstructive Surgery), and SFO (Société Francaise d’Ophtalmologie) allow for the diffusion of knowledge about ophthalmic malignancies, including biobanks. 

### 5.8. Education and Training

A Master in Science (MSc) degree entitled “Biobanks and complex data management” has been set up by the Côte d’Azur University (Nice, France). Developed by recognized experts in the field, this program is the first European MSc degree in the field offered by a public university (https://univ-cotedazur.eu/msc/biobanks-complex-data-management, accessed on 1 January 2023). Dedicated to Biological Sciences, this unique program is intended for students aiming to acquire professional skills in order to provide evolving biobank services, meet regulatory and user requirements, and manage biobanks. 

The “Biobanks and Complex Data Management” MSc allows international exchanges through a wide network of leading academic and industry partners.

## 6. Strengths of the NOMA Biobank

The strengths of our biobank are as follows:

(i) The NOMA biobank is certified (ISO 9001, NF-96S-900) and accredited (ISO 20387) and belongs to an already well-established biobank (Cote d’Azur Biobank) (www.biobank-cotedazur.fr, accessed on 1 January 2023),
(ii) It is stored in a university pathology laboratory (LPCE, Nice) accredited for clinical and molecular pathology according to the ISO 15189 standard,
(iii) Clinical, imaging, histological, biological, and genetic data are collected for each patient,
(iv) Its business model is supported by public funding programs,
(v) It has allowed for an increase in the amount of translational research conducted,
(vi) It allows for an increase in the number of scientific publications and national and international collaborations,
(vii) A master’s degree entitled “Biobanks and Complex Data Management” has been set up by the Côte d’Azur University (Nice, France) to train students to become biobankers.

## 7. Limitations

However, the NOMA Biobank also has several limitations. First, its small sample size could be explained by the fact that both UM and CM are rarely found in daily clinical practice. In addition, as already mentioned previously, a tissue biopsy is not routinely performed in UM. Despite this, this dedicated ophthalmic biobank is larger than other published ocular malignancy biobanks [9]. Second, this biobank does not currently include metastases, in particular, liver metastases in UM. Also, there is no retinoblastoma samples included in the biobank because the treatment is centralized at the Curie Institute in Paris. Third, there are high numbers of patients with a poor prognosis and aggressive UM (most tumors classified as pT3, high rate of extraocular extent, loss of chromosome 3, most tumors classified as Trolet 2b and 2c). This could be explained by the fact that more advanced UM are better managed with enucleation rather than with proton therapy. This could limit the possibilities of translational research in early UM stages. Fourth, samples are mainly collected at the time of initial tumor management and the collection is not repeated during the follow-up, thus limiting the ability of our biobank to monitor treatment response. Finally, a common challenge for most biobanks is the difficulty of synthesizing all clinical, histological, biological, and genetic data in a single database. In our case, clinical and imaging data are stored in one database (Synapse), while biological, histological, and genetic data are stored in another database. 

## 8. Conclusions

Dedicated, secured, and accredited biobanks are essential to collect high-quality samples to promote translational and multicentric ocular oncology research with the final aim of offering customized medicine. This is especially true for ophthalmic malignancies due to their scarcity and the lack of tissue biopsies performed in intraocular malignancies. Setting up such biobanks is time-, staff-, and cost-consuming and should be financially supported. The existing biobanks need to be promoted and new biobanks need to be created to develop outstanding international collaborations. 

## Figures and Tables

**Table 1:** Incidence of the main ophthalmic malignancies.

Tumor Histology	Incidence per Million Inhabitants (Reference)
UM	6 [2]
CM	0.8 [20]
Conjunctival squamous cell carcinoma	0.3 [22]
Primary eyelid melanoma	1 [23]

UM: uveal melanoma; CM: conjunctival melanoma.

**Table 2:** Summary of the NOMA Biobank sample processing (all ocular tumors).

Year	Solid Biopsy Samples	Liquid Biopsy Samples	AH Puncture Samples
2013	4	0	0
2014	5	0	0
2015	40	0	0
2016	131	0	0
2017	87	0	0
2018	94	710	0
2019	152	188	0
2020	97	307	11
2021	118	870	14
2022	108	647	14

**Table 3:** Summary of collected samples in terms of dedicated processing, amount, and storage conditions. (*) The paraffin protocol has been previously described [41]; (**) two consecutive centrifugations at 2000× *g* at 4 °C; (***) first centrifugation at 1000× *g* for 20 min in a dedicated Ficoll tube, followed by a second centrifugation at 400× *g* for 10 min at room temperature. FFPE: formalin-fixed paraffin-embedded. PBMC: peripheral blood mononuclear cells.

Type of Sample	Processing	Amount	Storage Condition
Tissue			
Frozen tumor tissue	None	5–50 mg	−80 °C
Frozen healthy choroid	None	5–50 mg	−80 °C
Paraffin-embedded tumor tissue	Paraffin protocol (*)	1–5 FFPE blocks	4 °C
Paraffin-embedded healthy choroid	Paraffin protocol (*)	1 FFPE blocks	4 °C
Blood			
Whole blood	None	1 mL per aliquot	−80 °C
Plasma	Centrifugation (**)	1 mL per aliquot	−80 °C
PBMC	Centrifugation (***)	0.5–10 million per 1-mL aliquot	−196 °C (liquid nitrogen)
Aqueous humor	None	10–50 µL per aliquot	−80 °C

**Table 4:** Summary of the data collected in the dedicated SYNAPSE database.

Demographics	Clinical and Radiological Data	Tumor-Related Radiological Data	Histological Data	Genetic Data
Gender Age Personal and family history of cancer	Visual acuity Intraocular pressure Cataract Retinal detachment Optic nerve involvement Extraocular extent Intravitreal injections Follow-up	Diameter Thickness Ciliary body involvement	pTNM classification Cell type (epithelioid, fusiform, mixed) Mitoses Extraocular extent Necrosis Inflammatory infiltration Optic nerve involvement BAP 1 status Extent of resection (R0, R1, R2)	Chromosomal abnormalities Trolet classification

**Table 5:** Summary of the data collected in the LPCE database (for all tumors except UM).

Demographics	Histological Data
Age Gender Date of surgery or collection	Histological diagnosis pTNM classification of the relevant tumor Tumor size % of tumor cells Cold ischemia time Primary vs. recurrent tumor Inflammatory infiltrate Ulceration Mitotic index Extent of resection (R0, R1, R2)

**Table 6:** Detailed composition of the NOMA Biobank samples.

Tumor Location	Histological Subtype: Number (%)	Tissue Biopsy: Number (%)	Venous Liquid Biopsy: Number (%)
Intraocular N = 160	Uveal melanoma: 160 (100)	66 (41)	124 (77.5)
Conjunctival N = 31	Conjunctival melanoma: 21 (68) Conjunctival naevus: 8 (25.5) Squamous cell carcinoma: 2 (6.5)	31 (100)	11 (35.5)
Orbit N = 16	Lymphoma: 8 (50) Carcinoma: 4 (25) Schwanoma: 3 (19) Solitary fibrous tumor: 1 (6)	16 (100)	0 (0)

**Table 7:** Histological features of enucleated UM patients. Cold ischemia time (time between resected specimen collection in the operative room and the freezing procedure); * n = 40 patients.

Number of Enucleated Patients (%)	66 (100)
Primary Enucleation	62 (94)
Secondary Enucleation	4 (6)
Tumor thickness in mm: mean (range)	12.1 (0.2–20)
Epithelioid subtype: number (%)	44 (67)
Extraocular extent: number (%)	13 (19.7)
Ciliary body infiltration: number (%)	22 (33)
Mitoses/mm^2^: mean number (range)	1.9 (0–21)
% of tumor cells in frozen sample: mean (range)	75.2 (0–95)
Cold ischemia time in min: mean (range)	34.8 (15–180)
BAP 1 loss: number (%) *	30 (75)

**Table 8:** Current national and international collaborations.

Institution	Type of Collaboration (Recent Related Articles)
Oncology Department, Antoine Lacassagne Cancer Centre, Nice, France	Fundamental and clinical [1,26,52,53,54]
Team 1, Molecular Mediterranean Medicine Centre (C3M), Nice, France	Fundamental [50,51,55,56]
Ophthalmology Department, Lyon University Hospital, France	Clinical [57]
Oculoplastic Department, Jules Gonin Eye Hospital, Lausanne, Switzerland	Clinical [52,58,59]
Anatomic Pathology Service, Pathology Department, Centro Hospitalar e Universitário do Porto, Portugal	Fundamental [6]
Liverpool Ocular Oncology Research Department, United Kingdom	Fundamental
